# Safety and Early Clinical Outcomes of Sacroiliac Fusion for Sacral Fractures via a Modified S1 Corridor: An Exploratory Comparison of Triangular and Threaded 3D-Printed Titanium Implants

**DOI:** 10.3390/medicina62081547

**Published:** 2026-08-12

**Authors:** Franz-Joseph Dally, Joe Mehanna, Peter Fennema, Marcus Rickert, Sascha Gravius, Frederic Bludau, Steffen Heinrich Schulz

**Affiliations:** 1 Orthopaedic and Trauma Surgery Center, University Medical Center Mannheim, 68167 Mannheim, Germany; franz.dally@umm.de (F.-J.D.); joe.mehanna@umm.de (J.M.); sascha.gravius@umm.de (S.G.); frederic.bludau@umm.de (F.B.); 2 AMR Advanced Medical Research GmbH, 8708 Männedorf, Switzerland; peter.fennema@amr-cro.com; 3 Orthopädie Rickert, 63500 Seligenstadt, Germany; rickert@dr-marcus-rickert.de

**Keywords:** fragility fractures of the sacrum, fragility fractures of the pelvis, sacroiliac fusion, modified S1 corridor, 3D-printed titanium implants, iFuse™, iFuse TORQ™, Oswestry Disability Index

## Abstract

*Background and Objectives*: Fragility fractures of the sacrum are increasingly recognized in elderly osteoporotic patients and can cause persistent pain, immobility, and progressive pelvic ring instability. We previously described a modified S1 corridor targeting the higher-density cranial–ventral S1 vertebral body for safe percutaneous implant placement. This study reports the clinical outcomes of osteoporotic and pathologic sacral fractures treated through this corridor and presents an exploratory comparison of two 3D-printed titanium sacroiliac fusion implants: the triangular iFuse™ Implant System and the threaded iFuse TORQ™ (SI-BONE, Inc., Santa Clara, CA, USA). *Materials and Methods*: This retrospective single-center study analyzed 58 patients with fragility or pathologic sacral fractures (FFP IIIb–V; OF 2–5) treated via the modified S1 corridor between January 2021 and March 2026. Of these, 27 patients received triangular iFuse™ implants, and 31 patients received threaded iFuse TORQ™ implants. The primary outcome was procedural safety (implant revision, neurological injury, cortical breach). Secondary outcomes included the Oswestry Disability Index (ODI), Visual Analog Scale (VAS) for pain, operative time, and need for additional fixation. Group comparisons used non-parametric tests; multivariable linear regression adjusted for age, fracture severity (OF classification), and additional fixation. *Results*: At a mean follow-up of 17.8 ± 9.1 months (iFuse) and 4.1 ± 2.1 months (TORQ), no implant revisions, clinically significant cortical breaches, or neurological injuries were observed in either group. Patient-reported outcome data were available for 48 of 58 patients (20 iFuse, 28 TORQ). Patients in the iFuse™ group were older (80.9 ± 8.5 vs. 76.0 ± 9.8 years; *p* = 0.050), and OF classification differed between groups, with a higher proportion of OF 4 fractures in the TORQ™ group (*p* = 0.003). After adjustment for age, OF classification, and additional fixation, TORQ™ implantation was associated with a 15.6-point lower postoperative ODI (*p* < 0.001), a 13.5-point greater ODI improvement (*p* < 0.001), a 0.94-point lower postoperative VAS (*p* = 0.006), and a 1.36-point greater VAS reduction (*p* < 0.001). Findings remained significant in a sensitivity analysis restricted to patients without additional fixation. *Conclusions*: Sacroiliac fusion via a modified density-oriented S1 corridor was safe for both large implant types, with no revisions in the analyzed cohort. In this retrospective, non-randomized cohort, threaded iFuse TORQ™ implants were associated with better patient-reported outcomes after adjustment for baseline imbalances. Because the groups were not randomized and follow-up duration differed substantially, these comparative findings are hypothesis-generating and require confirmation in prospective, controlled studies.

## 1. Introduction

The increasing incidence of fragility fractures of the pelvis (FFP) in aging populations represents a substantial clinical challenge that has been described as a “silent epidemic” [1]. Sacral insufficiency fractures, a subset of FFP, result from normal physiological loading on osteoporotic bone and can cause severe pain, loss of mobility, and progressive dependency [2,3]. Unlike high-energy pelvic injuries in younger patients, insufficiency fractures may follow a dynamic “instability cascade” in which an initial unilateral fracture leads to altered load transmission, contralateral involvement, and progressive bilateral instability [4].

While conservative management with analgesia and protected weight-bearing remains the initial treatment approach, usually for five to seven days, a substantial proportion of patients fail to mobilize adequately and remain immobilized by pain [5,6]. Prolonged immobility in elderly patients is associated with significant complications including pneumonia, venous thromboembolism, pressure ulcers, and deconditioning, all of which contribute to elevated morbidity and mortality [7]. Consequently, surgical stabilization has been increasingly advocated when conservative treatment fails, with the primary goal of enabling early weight-bearing and mobilization [6,8].

Percutaneous iliosacral screw fixation is widely used for posterior pelvic ring stabilization [9]. However, in osteoporotic bone, conventional screws are associated with high rates of loosening and backing out—reported in up to 20% of cases—owing to limited purchase in the low-density trabecular bone of the sacral ala [10,11]. Morphometric analyses have identified a distinct zone of reduced bone mineral density in the sacral ala, termed the “alar void,” which represents a region of particular vulnerability for fixation failure [12,13]. In contrast, the cranial and ventral portion of the S1 vertebral body—the promontory region—retains comparatively higher bone density even in osteoporotic patients [12,13].

Based on these anatomical and densitometric findings, our group developed a modified S1 corridor technique that redirects the fixation trajectory from a more posterior and caudal iliac entry point cranially and anteriorly toward the S1 promontory, deliberately bypassing the low-density alar region [14]. We previously demonstrated the safety and technical feasibility of this corridor for large-diameter 3D-printed titanium sacroiliac fusion implants under conventional 2D fluoroscopic guidance [14]. Two such implant systems are currently available from the same manufacturer (SI-BONE Inc., Santa Clara, CA, USA): the iFuse Implant System, which has a triangular cross-section and porous titanium surface designed for interference fit and rotational resistance, and the iFuse TORQ, a threaded cylindrical implant enabling active lag compression across the sacroiliac joint while offering improved rotational stability over conventional screws based on its 3D-printed thread layout [15].

Although the iFuse™ has been extensively evaluated for sacroiliac joint dysfunction, with Level I evidence from two randomized controlled trials [16,17] and supportive long-term prospective data [18,19], no published clinical study has compared the triangular iFuse™ and threaded iFuse TORQ™ implants in a sacral fracture population, and no clinical outcome data have been reported for the TORQ™ implant in any fracture indication. This evidence base, however, concerns sacroiliac joint dysfunction—a degenerative, pain-driven condition distinct from sacral fragility fractures—and cannot be extrapolated to the fracture setting.

The purpose of this study was to report the safety and clinical outcomes of fragility and pathologic sacral fractures treated via the modified S1 corridor and to perform an exploratory comparison of patient-reported outcomes between triangular iFuse and threaded iFuse TORQ implants. We hypothesized that both implant types would demonstrate acceptable safety profiles and that threaded TORQ implants would show favorable signals in short-term functional recovery.

## 2. Materials and Methods

### 2.1. Study Design and Ethics

This was a retrospective, single-center cohort study conducted at the Orthopaedic and Trauma Surgery Center of the University Medical Center Mannheim, Germany. The study was approved by the Institutional Review Board of Ethics Committee II of the Medical Faculty Mannheim, University of Heidelberg (Protocol Code 2025-864_1-AF 24, amended on 12 December 2025 after initial approval on 6 December 2021 under approval code 2021-897). Informed consent was obtained from all participants.

### 2.2. Patient Selection

All consecutive patients who underwent percutaneous sacroiliac fusion via the modified S1 corridor between January 2021 and March 2026 were identified from the institutional surgical database. Patients were included if they underwent the procedure for a fracture of the sacrum, irrespective of underlying etiology (osteoporotic insufficiency, fragility fracture of the pelvic ring, pathologic or radionecrotic fracture, or complex injury including lumbosacral dissociation). Fracture classification was determined from preoperative CT imaging and clinical documentation using both the FFP classification [2] and the Osteoporotic Fractures of the Pelvis (OF-Pelvis) classification [8]. The cohort therefore included fractures across FFP grades IIIb through V and OF grades 2 through 5. Patients undergoing the procedure for primary sacroiliac joint arthritis, sacroiliac joint dysfunction, symphyseal disorders, or pelvic tumor resection as the principal indication were excluded.

### 2.3. Implant Selection

Implant selection was not randomized. The triangular iFuse™ Implant System (SI-BONE, Inc., Santa Clara, CA, USA) was used from the beginning of the study period (2021), while the iFuse TORQ™ (SI-BONE, Inc., Santa Clara, CA, USA) became available and was introduced into clinical practice during the study period. Both implants are 3D-printed titanium devices designed for sacroiliac fusion. The iFuse™ features a triangular cross-section (up to 14.2 mm outer diameter) with a porous surface designed for interference fit and rotational resistance. The iFuse TORQ™ features a threaded cylindrical design (up to 13.5 mm outer diameter) that achieves active lag compression across the sacroiliac joint and provides self-harvesting of bone graft through its fenestrated structure [15].

### 2.4. Surgical Technique

The surgical technique has been described in detail in our companion technical paper [14]. Briefly, all patients underwent preoperative CT of the pelvis with multiplanar reconstruction to define a modified S1 corridor targeting the cranial–ventral S1 vertebral body (promontory). The planned trajectory originated from a more posterior and caudal iliac entry point compared with the standard vestibular trajectory. Surgery was performed with the patient in the prone position under general anesthesia using conventional 2D fluoroscopy. A true lateral view with superimposition of the greater sciatic notches served as the primary fluoroscopic landmark. A 3.2 mm guidewire was advanced manually with a hammer to provide tactile feedback, followed by predrilling and implant insertion. Intraoperative 3D imaging (cone-beam CT) was used in selected cases to verify osseous containment. Patients were permitted weight-bearing as tolerated immediately postoperatively.

### 2.5. Outcome Measures

The primary outcome was procedural safety, assessed by the occurrence of implant revision, clinically significant implant malposition (defined as implant malposition, cortical breach, or neural-structure proximity that required operative intervention or produced attributable symptoms), and iatrogenic neurological or vascular injury. Postoperative CT was used to evaluate implant position and osseous containment. Implant proximity to the neuroforamen and cortical breach were graded radiographically as no cortical breach, less than 2 mm breach, 2–5 mm breach, or greater than 5 mm breach. All patients underwent postoperative CT of the pelvis using the institution’s standard multiplanar protocol; implant position and cortical breach were graded on these scans, and radiographic assessments were not performed by independent blinded reviewers.

Secondary outcomes included the Oswestry Disability Index (ODI, 0–100%, with higher scores indicating greater disability) and the Visual Analog Scale for pain (VAS, 0–10) assessed preoperatively and at the most recent postoperative follow-up visit. Additional variables recorded included operative time, number of implants, need for additional fixation (e.g., transiliac–transsacral internal fixation, spinopelvic fixation, screw osteosynthesis, kyphoplasty, or anterior ring stabilization), fracture classification (FFP and OF-Pelvis), and patient demographics.

### 2.6. Statistical Analysis

Continuous variables are presented as mean ± standard deviation. Categorical variables are presented as count and percentage. Between-group comparisons used the Mann–Whitney U test for continuous variables and Fisher’s exact test for categorical variables. Multivariable linear regression with robust standard errors was used to estimate the adjusted association between implant type and clinical outcomes, controlling for age, OF classification (OF 3 vs. OF 4), and the presence of additional fixation. A prespecified sensitivity analysis was conducted restricted to patients who did not receive additional fixation. All analyses were performed in Stata 15.1 (StataCorp, College Station, TX, USA). A two-sided *p*-value of less than 0.05 was considered statistically significant. The multivariable model was specified a priori and intentionally limited to the implant-group term and three adjustment covariates (age, OF classification, and additional fixation), the principal measured baseline imbalances; with four model terms and 46 patients, this provides approximately 11.5 observations per term. Model assumptions (linearity, homoscedasticity, and normality of residuals) were checked graphically, and collinearity was assessed using variance-inflation factors, with no material violations. All outcome analyses used complete cases without imputation. Given the sample size and the non-randomized design, the adjusted analyses are regarded as exploratory rather than confirmatory.

## 3. Results

### 3.1. Cohort Characteristics

Between January 2021 and March 2026, 58 consecutive patients underwent percutaneous sacroiliac fusion via the modified S1 corridor for a sacral fracture and met the inclusion criteria. Of these, 27 (46.6%) received triangular iFuse™ implants and 31 (53.4%) received threaded iFuse TORQ™ implants (Table 1). The majority of fractures were osteoporotic fragility fractures of the pelvic ring (FFP IIIb–V); the cohort also included a small number of pathologic and radionecrotic sacral fractures and two cases of lumbosacral dissociation with spinopelvic instability. Posterior involvement was bilateral in the large majority of patients in both groups (iFuse™ 24/27, 88.9%; TORQ™ 30/31, 96.8%; *p* = 0.34). Patient sex was not recorded in the study database.

Patients in the iFuse™ group were older than those in the TORQ™ group (80.9 ± 8.5 vs. 76.0 ± 9.8 years; *p* = 0.050). There were no significant differences in operative time (31.9 ± 11.3 vs. 35.2 ± 12.5 min; *p* = 0.325) or in the number of implants placed per patient (1.93 ± 0.38 vs. 2.00 ± 0.26; *p* = 0.384). Additional fixation was performed in 8 iFuse™ patients (29.6%) and 8 TORQ™ patients (25.8%), without a statistically significant difference (*p* = 1.000). Lumbopelvic or spinopelvic fixation specifically was used in 4 iFuse™ patients (14.8%) and 4 TORQ™ patients (12.9%).

FFP classification data were available for 24 of 27 iFuse™ patients and all 31 TORQ™ patients; OF classification was available for all 58 patients. According to the OF-Pelvis classification, fracture severity differed between groups, with a higher proportion of OF 4 fractures in the TORQ™ group (27/31, 87.1%) than in the iFuse™ group (14/27, 51.9%; *p* = 0.003 for the overall OF distribution, Table 1). According to the FFP classification, the overall distribution did not differ significantly between groups (*p* = 0.092), although FFP IVb represented the majority in both groups (14/24, 58.3% in the iFuse™ group vs. 26/31, 83.9% in the TORQ™ group).

Mean follow-up was 17.8 ± 9.1 months in the iFuse group and 4.1 ± 2.1 months in the TORQ group (*p* < 0.001). The TORQ group had shorter follow-up, reflecting its later introduction into clinical practice.

### 3.2. Safety Outcomes

No implant revisions were required in either group and no iatrogenic neurological or vascular injuries were documented. On postoperative CT assessment, no cortical breach was observed in 16 of 27 iFuse™ patients (59.3%) and 24 of 31 TORQ™ patients (77.4%); a breach of less than 2 mm in 7 iFuse™ and 7 TORQ™ patients (25.9% vs. 22.6%); a 2–5 mm breach in 2 iFuse™ patients (7.4%) and no TORQ™ patients; and a breach greater than 5 mm in 2 iFuse™ patients (7.4%) and no TORQ™ patients. Categories reflect the worst cortical breach observed across implants at the patient level. None of these radiographic findings required operative revision or was associated with clinically significant symptoms. One iFuse™ patient (3.7%) had a radiographic finding recorded as clinically significant (implant proximity causing local discomfort without neurological deficit, managed conservatively); no corresponding event occurred in the TORQ™ group (*p* = 0.466). Implant proximity to the S1 neuroforamen was more frequent in the iFuse™ group (7 patients, 25.9%) than in the TORQ™ group (1 patient, 3.2%) (*p* = 0.020), and none of these findings were symptomatic. Proximity to the L5 or S1 nerve roots was documented in 10 of 26 evaluable iFuse™ patients (38.5%) and 6 of 31 TORQ™ patients (19.4%), without reaching statistical significance (*p* = 0.144) and without associated neurological deficit in any case.

### 3.3. Clinical Outcomes

Preoperative ODI and VAS scores were comparable between groups (ODI: 74.7 ± 5.1 vs. 74.8 ± 8.0, *p* = 0.659; VAS: 7.50 ± 0.83 vs. 7.89 ± 0.88, *p* = 0.141). Both groups demonstrated substantial improvements in patient-reported outcomes following surgery. Postoperative ODI scores were significantly lower in the TORQ™ group compared with the iFuse™ group (29.4 ± 10.0 vs. 42.9 ± 4.8; *p* < 0.001), and VAS pain scores were also lower (3.61 ± 0.96 vs. 4.40 ± 1.05; *p* = 0.017). The magnitude of improvement from baseline was significantly greater in the TORQ™ group for both ODI (−45.3 ± 11.7 vs. −31.8 ± 6.9; *p* < 0.001) and VAS (−4.29 ± 0.81 vs. −3.10 ± 0.85; *p* < 0.001) (Table 2). Paired patient-reported outcome data (both ODI and VAS) were available for 20 of 27 iFuse™ patients (74.1%) and 28 of 31 TORQ™ patients (90.3%), yielding a common analytic sample of 48 patients for all outcome comparisons. All outcome analyses used complete cases without imputation. Patients without paired outcome data (*n* = 10) were somewhat younger (72.8 vs. 79.4 years; *p* = 0.07) and had a lower proportion of OF 4 fractures (40% vs. 77%), but missing outcome data were not significantly associated with implant group (*p* = 0.16).

### 3.4. Adjusted Analyses

Given the baseline differences in age and OF classification, multivariable linear regression was performed adjusting for age, OF classification (OF 3 vs. OF 4), and presence of additional fixation. The adjusted analysis was restricted to patients with complete outcome and covariate data and OF of 3 or 4 (*n* = 46; 20 iFuse™, 26 TORQ™). After adjustment, TORQ™ implantation remained independently associated with improved outcomes across all measured endpoints: a 15.6-point lower postoperative ODI (95% CI −20.5 to −10.7; *p* < 0.001), a 13.5-point greater improvement in ODI (95% CI −20.2 to −6.8; *p* < 0.001), a 0.94-point lower postoperative VAS score (95% CI −1.59 to −0.28; *p* = 0.006), and a 1.36-point greater VAS reduction (95% CI −1.87 to −0.86; *p* < 0.001) (Table 3; full models in Supplementary Table S1).

### 3.5. Sensitivity Analysis

In a prespecified sensitivity analysis restricted to patients who did not receive additional fixation (*n* = 36 with complete outcome data and OF 3 or 4; 15 iFuse™ and 21 TORQ™), the observed between-group differences remained statistically significant and the effect sizes were, if anything, slightly larger than in the primary analysis. TORQ™ implantation continued to be associated with lower postoperative ODI (adjusted difference −16.5 points; *p* < 0.001), greater ODI improvement (−14.7 points; *p* < 0.001), lower postoperative VAS (−1.03 points; *p* = 0.011), and greater VAS reduction (−1.26 points; *p* < 0.001), confirming that the difference persisted in patients treated with the sacroiliac implants alone.

## 4. Discussion

To our knowledge, this study presents the first clinical comparison of large triangular and threaded 3D-printed titanium sacroiliac fusion implants for the treatment of fragility and pathologic sacral fractures through a modified density-oriented S1 corridor. Two main findings emerged from this analysis: both implant designs proved safe to deploy through this corridor, with no revisions required, and the threaded TORQ implants were associated with better patient-reported outcomes—both in absolute postoperative scores and in improvement from baseline—after adjustment for age, fracture severity, and additional fixation.

### 4.1. Procedural Safety

The absence of implant revisions and iatrogenic neurological injuries in this cohort is consistent with our prior technical safety series of 137 implant placements through the modified S1 corridor [14]. The zero revision rate compares favorably with reported screw loosening rates of up to 20% for conventional iliosacral screws in osteoporotic bone [10], although loosening does not always necessitate revision. This observation is likely attributable to two factors: the deliberate targeting of the higher-density S1 promontory region, which provides better bone purchase than the alar trajectory [12,13], and the use of large-diameter 3D-printed titanium implants (10.0–14.2 mm), which achieve greater initial fixation stability than standard 7.3 mm cannulated screws [15]. Cortical breaches greater than 2 mm were observed in four iFuse™ patients and no TORQ™ patients, and neuroforaminal proximity was more frequent in the iFuse™ group (25.9% vs. 3.2%); none of these radiographic findings required revision or produced neurological symptoms, but the pattern is consistent with the greater cross-sectional footprint of the triangular implant and warrants continued monitoring in future series. The safety of both implant types through this corridor supports the continued use of either design based on clinical indication and surgeon preference.

### 4.2. Patient-Reported Outcomes

Both implant groups demonstrated clinically meaningful improvements in disability and pain following surgery. Mean ODI improvement exceeded the commonly cited minimal clinically important change threshold of 10–15 points in both groups, reaching 31.8 points in the iFuse™ group and 45.3 points in the TORQ™ group [20,21]. Similarly, VAS improvements of 3.1 and 4.3 points exceeded the typical minimal clinically important change of 2 points for pain [21]. These reductions in disability and pain are consistent with the broader FFP surgical literature, in which operative stabilization has been associated with superior pain relief and earlier mobilization compared with conservative management [6,7,8].

The significantly greater improvement in the TORQ™ group persisted after adjustment for the principal baseline imbalances identified in this study: age (iFuse™ patients were on average five years older) and fracture severity (the TORQ™ group had a higher proportion of OF 4 fractures, which paradoxically represent more unstable patterns). Additional fixation rates were comparable between groups. The adjusted association remained large (TORQ™ coefficient −15.6 points for postoperative ODI) and the direction of the key baseline imbalances did not uniformly favor the TORQ™ group. Age was an independent predictor in the postoperative ODI, postoperative VAS, and VAS-change models, confirming that adjustment for this imbalance was substantively important rather than merely a technical exercise. Because implant type was confounded with calendar time in this sequential cohort, these comparative findings should be interpreted as exploratory associations rather than as evidence of device superiority.

### 4.3. Potential Mechanisms

Several mechanisms may underlie the observed differences. The threaded design of the TORQ implant generates active lag compression across the sacroiliac joint, which may reduce micromotion and the “windshield wiper” toggling effect seen with conventional fixation in osteoporotic bone. The conical head may further contribute to compression when seated against the iliac cortex. Chatain et al. demonstrated in a cadaveric model that iFuse TORQ implants achieved significantly higher insertion and removal torques at S1 compared with standard 7.3 mm lag screws, suggesting superior initial mechanical purchase [15]. The fenestrated structure of the TORQ implant additionally harvests local bone graft during insertion [15], potentially accelerating osseointegration. However, whether these biomechanical advantages translate directly into the clinical outcome differences observed in this study cannot be determined from the present data.

### 4.4. Comparison with the Published Literature

Our findings suggest that sacroiliac fusion through the modified S1 corridor represents an additional minimally invasive option, with short operative times (mean 32–35 min) and immediate full weight-bearing.

The only published comparison of triangular versus cylindrical threaded sacroiliac fusion implants is by Claus et al., who reported no significant difference in patient-reported outcomes between the two devices [22]. However, that study addressed sacroiliac joint dysfunction—not fractures—and used a different threaded implant system, limiting the applicability of those findings to the present population.

### 4.5. Limitations

This study has several limitations. First, the retrospective, non-randomized design introduces selection bias and confounding by indication. Implant selection was largely determined by temporal availability rather than a clinical algorithm, creating a sequential-cohort effect in which the iFuse group was treated earlier and the TORQ group later. This temporal sequence means that improvements in surgical technique, patient selection, and perioperative management over the study period may partially account for the observed differences. In effect, implant type is confounded with calendar time, so a genuine device effect cannot be separated from these secular trends. Second, the age difference (approximately 5 years) and the fracture severity imbalance (more OF 4 fractures in the TORQ group) represent important baseline inequalities. While the multivariable adjustment addresses these measured confounders, residual confounding from unmeasured variables—including bone mineral density, frailty, comorbidity burden, pre-injury functional status and mobility, osteoporosis pharmacotherapy, rehabilitation intensity, and patient sex—cannot be excluded.

Third, the sample size of 58 patients limits statistical power and the ability to perform robust subgroup analyses. Furthermore, paired patient-reported outcome data (both ODI and VAS) were unavailable for 7 of 27 iFuse™ patients (25.9%) and 3 of 31 TORQ™ patients (9.7%). The adjusted analyses were additionally restricted to patients with OF 3 or 4 (excluding 1 iFuse™ patient with OF 2 and 2 TORQ™ patients with OF 5), yielding a common analytic sample of 46 patients for the multivariable models. Nevertheless, attrition bias cannot be fully excluded. Fourth, the follow-up duration was not standardized, and the TORQ group does have shorter follow-up due to its later introduction; the clinical significance of the observed outcome differences at longer follow-up remains unknown. Moreover, because follow-up duration is almost entirely collinear with implant group, it cannot be meaningfully entered as a covariate, and patient-reported outcomes were recorded at the most recent visit rather than at standardized time points. Fifth, although the additional fixation rate did not differ significantly between groups, the types of adjunct procedures (spinopelvic fixation, anterior ring stabilization, kyphoplasty, screw osteosynthesis) were heterogeneous and may have differentially influenced recovery. Sixth, this is a single-center experience from a specialized team with extensive familiarity with this technique, and the results may not be generalizable to other surgical settings or teams without equivalent preoperative 3D planning workflows. Finally, the outcome assessors were not blinded to implant type.

These findings should be interpreted in light of the retrospective, non-randomized design and limited sample size. Although the association between TORQ implantation and improved outcomes remained significant after adjustment and sensitivity analysis, residual confounding cannot be excluded. Further comparative studies are needed to determine whether these differences persist in larger, more standardized cohorts.

## 5. Conclusions

Percutaneous sacroiliac fusion via a modified density-oriented S1 corridor was safe for both triangular and threaded 3D-printed titanium implants in patients with osteoporotic and pathologic sacral fractures, with no revisions in the analyzed cohort. Both implant types produced clinically meaningful improvements in disability and pain. In this retrospective comparative analysis, threaded iFuse TORQ™ implants were associated with better patient-reported outcomes after adjustment for age, fracture severity, and additional fixation. This association should be regarded as hypothesis-generating. Further comparative studies, preferably prospective and multicenter, are needed to determine whether these differences persist in larger cohorts and at longer follow-up.

## Figures and Tables

**Table 1:** Baseline and procedural characteristics of patients treated via the modified S1 corridor.

Variable	iFuse™ (*n* = 27)	TORQ™ (*n* = 31)	*p*-Value
Age, years	80.9 ± 8.5	76.0 ± 9.8	0.050
Surgery duration, min	31.9 ± 11.3	35.2 ± 12.5	0.325
Number of implants	1.93 ± 0.38	2.00 ± 0.26	0.384
Additional fixation, *n* (%)	8 (29.6%)	8 (25.8%)	1.000
Follow-up, months	17.8 ± 9.1	4.1 ± 2.1	<0.001
Bilateral involvement, *n* (%)	24 (88.9%)	30 (96.8%)	0.329
Fracture etiology			0.311
Osteoporotic insufficiency/FFP, *n* (%)	24 (88.9%)	24 (77.4%)	
Pathologic/tumor, *n* (%)	1 (3.7%)	1 (3.2%)	
Radionecrotic, *n* (%)	1 (3.7%)	3 (9.7%)	
Traumatic, *n* (%)	1 (3.7%)	1 (3.2%)	
Lumbosacral dissociation, *n* (%)	0 (0.0%)	2 (6.5%)	
OF classification			0.003
OF 2, *n* (%)	1 (3.7%)	0 (0.0%)	
OF 3, *n* (%)	12 (44.4%)	2 (6.5%)	
OF 4, *n* (%)	14 (51.9%)	27 (87.1%)	
OF 5, *n* (%)	0 (0.0%)	2 (6.5%)	
**FFP classification**			0.092
FFP IIIb, *n* (%)	4 (16.7%)	2 (6.5%)	
FFP IIIc, *n* (%)	5 (20.8%)	1 (3.2%)	
FFP IVb, *n* (%)	14 (58.3%)	26 (83.9%)	
FFP IVc, *n* (%)	1 (4.2%)	0 (0.0%)	
FFP V, *n* (%)	0 (0.0%)	2 (6.5%)	

Continuous variables presented as mean ± standard deviation and compared using Mann–Whitney U test; categorical variables compared using Fisher’s exact test. The fracture-etiology *p*-value compares osteoporotic/FFP versus all other etiologies; laterality denotes bilateral versus unilateral posterior involvement. Patient sex was not recorded in the database. OF: osteoporotic fractures.

**Table 2:** Patient-reported outcomes in the study cohort.

Variable	iFuse™ (*n* = 20)	TORQ™ (*n* = 28)	*p*-Value
ODI preoperative	74.7 ± 5.1	74.8 ± 8.0	0.659
ODI postoperative	42.9 ± 4.8	29.4 ± 10.0	<0.001
ODI change	−31.8 ± 6.9	−45.3 ± 11.7	<0.001
VAS preoperative	7.50 ± 0.83	7.89 ± 0.88	0.141
VAS postoperative	4.40 ± 1.05	3.61 ± 0.96	0.017
VAS change	−3.10 ± 0.85	−4.29 ± 0.81	<0.001

Presented as mean ± standard deviation. ODI, Oswestry Disability Index (0–100%); VAS, Visual Analog Scale (0–10). Postoperative values compared using Wilcoxon rank-sum test.

**Table 3:** Adjusted association between implant type and clinical outcomes (multivariable linear regression).

Outcome	TORQ Coefficient	95% CI	*p*-Value
ODI postoperative	−15.63	−20.51 to −10.75	<0.001
ODI change	−13.48	−20.20 to −6.77	<0.001
VAS postoperative	−0.94	−1.59 to −0.28	0.006
VAS change	−1.36	−1.87 to −0.86	<0.001

Coefficients represent the adjusted difference associated with TORQ relative to iFuse. Model adjusted for age (continuous), OF classification (OF 3 vs. OF 4), and additional fixation (yes/no). Robust standard errors. *n* = 46 patients with complete outcome and covariate data and OF 3 or 4 (20 iFuse™, 26 TORQ™). Full models are reported in Supplementary Table S1.

## Data Availability

The data presented in this study are available on request from the corresponding author. The data are not publicly available due to patient privacy.

## Supplementary Materials

The following supporting information can be downloaded at: https://www.mdpi.com/article/10.3390/medicina62081547/s1, Table S1. Full multivariable linear regression models for patient-reported outcomes.
