# Supplementary material for: Safety and Early Clinical Outcomes of Sacroiliac Fusion for Sacral Fractures via a Modified S1 Corridor: An Exploratory Comparison of Triangular and Threaded 3D-Printed Titanium Implants
Source: Medicina (Kaunas). 2026 Aug 12;62(8):1547. doi: 10.3390/medicina62081547 (PMC13515031; doi:10.3390/medicina62081547)
Supplement: Supplementary file 1 [file medicina-62-01547-s001.zip › medicina-4342960-supplementary.pdf]

Supplementary Material

Table S1. Full multivariable linear regression models for patient-reported outcomes.

Covariate	Coefficient	95% CI	p-value
**Panel A. Postoperative ODI**			
iFuse TORQ™ (vs iFuse™)	−15.63	−20.51 to −10.75	<0.001
Age (per year)	−0.33	−0.61 to −0.05	0.021
OF 4 (vs OF 3)	1.62	−3.30 to 6.54	0.510
Additional fixation (yes vs no)	3.60	−2.53 to 9.74	0.242
Constant	68.06	45.16 to 90.96	<0.001
**Panel B. ODI change from baseline**			
iFuse TORQ™ (vs iFuse™)	−13.48	−20.20 to −6.77	<0.001
Age (per year)	−0.09	−0.40 to 0.23	0.570
OF 4 (vs OF 3)	−1.86	−8.36 to 4.65	0.567
Additional fixation (yes vs no)	5.93	−2.67 to 14.53	0.171
Constant	−24.73	−50.58 to 1.12	0.060
**Panel C. Postoperative VAS**			
iFuse TORQ™ (vs iFuse™)	−0.94	−1.59 to −0.28	0.006
Age (per year)	−0.034	−0.064 to −0.005	0.025
OF 4 (vs OF 3)	0.01	−0.96 to 0.97	0.988
Additional fixation (yes vs no)	−0.14	−0.89 to 0.62	0.714
Constant	7.24	4.93 to 9.55	<0.001
**Panel D. VAS change from baseline**			
iFuse TORQ™ (vs iFuse™)	−1.36	−1.87 to −0.86	<0.001
Age (per year)	−0.031	−0.056 to −0.005	0.020
OF 4 (vs OF 3)	0.02	−0.65 to 0.69	0.947
Additional fixation (yes vs no)	−0.37	−1.02 to 0.27	0.251
Constant	−0.48	−2.58 to 1.61	0.643

Each panel is a separate multivariable linear regression model (ordinary least squares with heteroscedasticity-consistent [HC1] robust standard errors). The analytic sample comprised patients with complete paired ODI and VAS data and OF classification 3 or 4 (n = 46; 20 iFuse™, 26 TORQ™). The reference categories are iFuse™ for implant type, OF 3 for fracture severity, and no additional fixation. ODI, Oswestry Disability Index (0–100%); VAS, Visual Analog Scale (0–10); OF, Osteoporotic Fractures of the Pelvis classification. Negative coefficients for the iFuse TORQ™ term indicate lower postoperative scores or greater improvement (i.e., better outcomes) relative to iFuse™. Estimates correspond to the models reported in Table 3 of the main text.
